# A case of *Listeria monocytogenes* endophthalmitis with recurrent inflammation and novel management

**DOI:** 10.1186/s12348-015-0058-8

**Published:** 2015-10-05

**Authors:** Adam C. Weber, Ashleigh L. Levison, Sunil K. Srivastava, Careen Y. Lowder

**Affiliations:** Cole Eye Institute, Cleveland Clinic Foundation, 9500 Euclid Avenue, Mail Code i-32, Cleveland, OH 44195 USA

**Keywords:** *Listeria monocytogenes*, Bacterial endophthalmitis, Recurrent inflammation, Anterior chamber washout

## Abstract

**Background:**

*Listeria monocytogenes* is a rare cause of endogenous endophthalmitis. In the limited number of reported *Listeria* endophthalmitis cases, visual acuity outcomes have been very poor.

**Findings:**

Here, we report a case of *Listeria* endophthalmitis that was complicated by recurrent inflammation. The patient required treatment with both intravitreal and long-term systemic antibiotics. An anterior chamber washout was necessary for the patient to regain 20/20 visual acuity.

**Conclusions:**

This case highlights the importance of considering *Listeria* early in the disease course, as it has low sensitivity to standard empiric antibiotic therapy. It also stresses the importance of addressing damaging inflammation in infectious conditions.

## Findings

### Background

*Listeria monocytogenes* is a gram-positive bacillus transmitted through contaminated food including unpasteurized dairy products, meats, seafood, and raw vegetables. It is typically associated with gastrointestinal illness and rarely affects the eye [1]. The most common form of ocular listeriosis in adults is conjunctivitis, although keratitis, endophthalmitis, and chorioretinitis have also been described [2–5]. *Listeria* endogenous endophthalmitis has been described in both immunocompetent and immunocompromised patients [2, 6]. While there are few *L. monocytogenes* endophthalmitis cases in the literature, Jackson et al. isolated *L. monocytogenes* in 10 of 267 endogenous endophthalmitis cases [7].

*L. monocytogenes* endophthalmitis typically presents with eye pain, increased intraocular pressure (IOP), and a fibrinous anterior chamber reaction. Many cases develop an evolving dark hypopyon due to iris pigment dispersion [8]. Most reports have final vision worse than 20/200, and only two reports document final visual acuity better than 20/60 [6, 9, 10]. We present a case of endogenous *L. monocytogenes* endophthalmitis in a healthy male who recovered 20/20 vision after aggressive intravitreal and systemic antibiotic therapy and anterior chamber washout. His follow-up course was complicated by recurrent inflammation. To our knowledge, this is the first reported case of *Listeria* endophthalmitis complicated by recurrent inflammation. This case highlights the importance of early diagnosis and treatment with consideration of early surgical intervention following antibiotic therapy to produce a better visual outcome.

### Case report

A 58-year-old Caucasian man presented with increasing right eye redness and tearing for 2 days. The patient’s past medical history was significant for well-controlled type 2 diabetes mellitus. On initial clinical exam, visual acuity was 20/400 in the right eye and 20/20 in the left eye. His intraocular pressure was 51 mmHg in the right eye and 17 mmHg in the left eye. Slit-lamp examination of the right eye showed 3+ conjunctival injection, corneal edema, and a fibrinous reaction in the anterior chamber. His left eye exam was unremarkable.

**Fig. 1:**
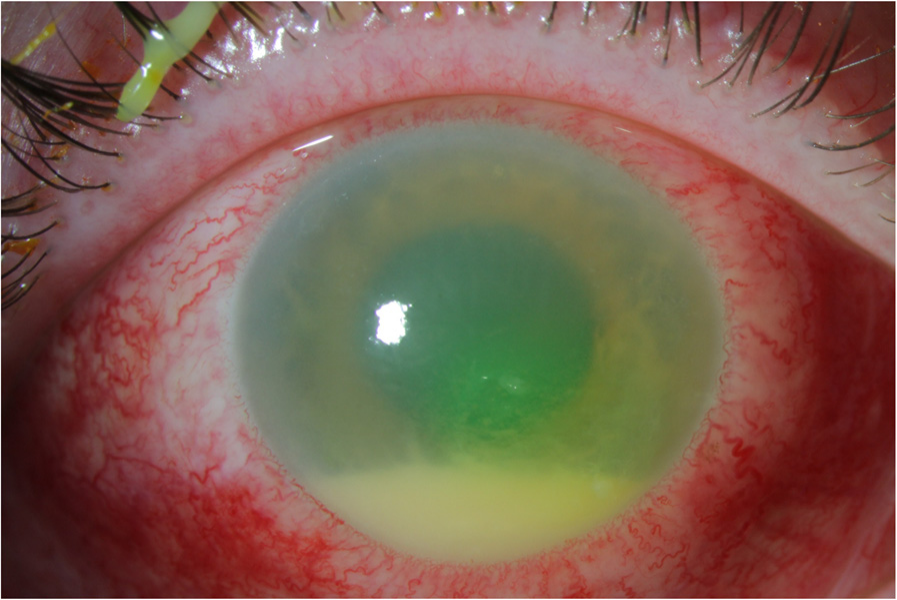
Photo documenting the development of a large hypopyon in the anterior chamber after having undergone a paracentesis

The patient’s right eye was treated with maximum medical therapy for the intraocular pressure but failed to respond. An anterior chamber paracentesis was performed to lower his intraocular pressure, and he was continued on maximum IOP-lowering agents. On follow-up the next day, visual acuity was 20/70 and intraocular pressure was 43 mmHg in the right eye. The remainder of the exam was unchanged. The patient was started on valacyclovir 1 g three times a day and topical prednisolone acetate for presumed herpetic keratouveitis. The following day, a paracentral epithelial defect and a 4-mm hypopyon was seen (Fig. 1). B-scan ultrasound showed anterior vitritis. Anterior chamber tap was performed and intravitreal vancomycin and ceftazidime were injected for suspected infectious endophthalmitis. The patient was started on fortified vancomycin and ceftazidime drops as well.

**Fig. 2:**
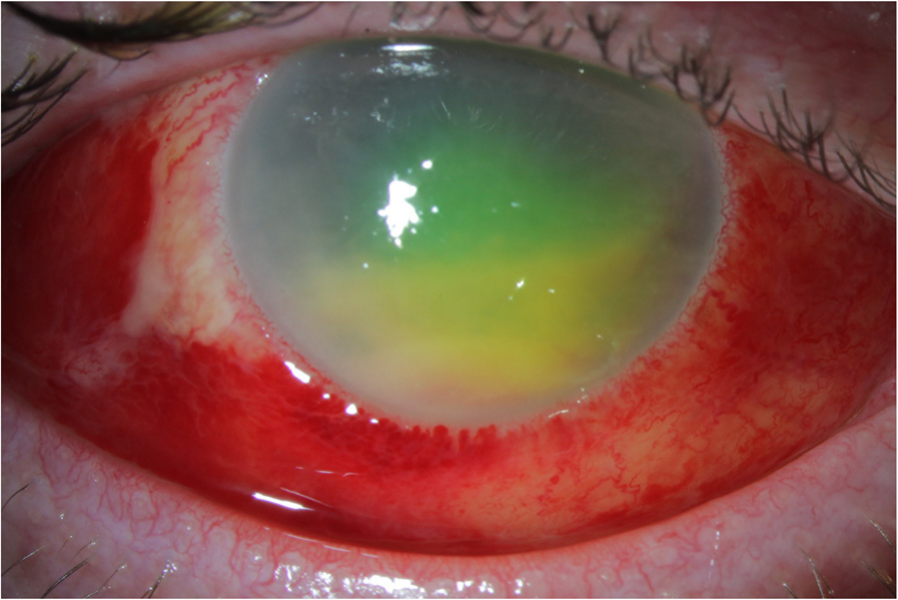
At follow-up after having undergone a tap and injection of vancomycin and ceftazidime, the hypopyon was larger. He was started on oral steroids

Over the next few days, the hypopyon increased in size and oral prednisone was started (Fig. 2). Culture of the anterior chamber tap grew *L. monocytogenes*. Intravitreal amikacin was given upon result of the anterior chamber tap due to poor coverage of vancomycin for *Listeria*. An infectious disease consult was obtained; systemic antibiotic therapy was initiated including a 1-month course of intravenous 3 g ampicillin every 6 h and oral double-strength trimethoprim-sulfamethoxazole twice a day. Oral steroids were stopped, and fortified antibiotic drops were changed to topical gentamicin.

**Fig. 3:**
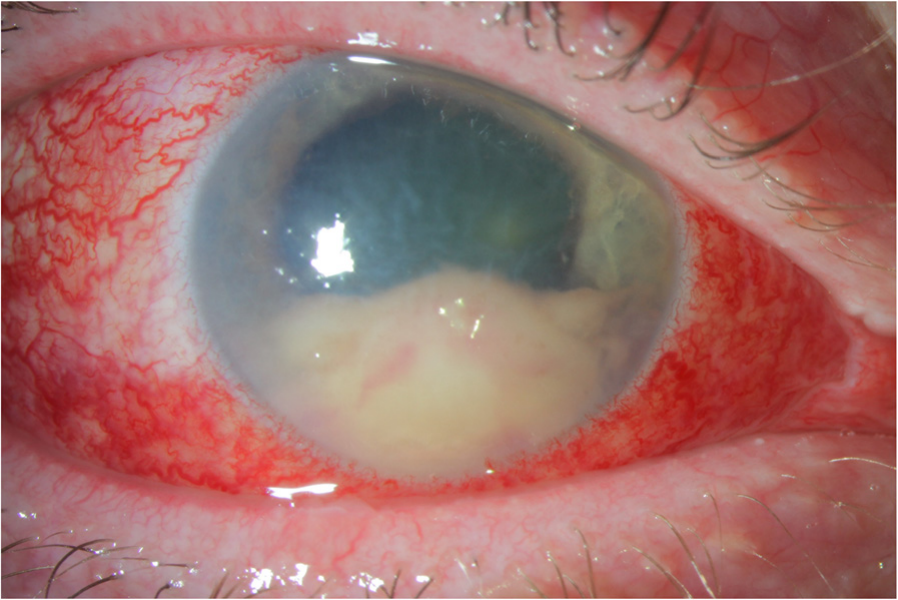
After improvement in both the infection and his vision, the hypopyon had organized into a consolidated dense mass. He underwent anterior chamber washout

The patient’s vision and exam slowly improved. The hypopyon, however, consolidated into a dense mass (Fig. 3); an anterior chamber washout was performed 2 weeks after initial presentation due to progressive corneal edema and lack of resolution. His ocular antihypertensives and steroids were slowly tapered. Four weeks status post anterior chamber washout, his visual acuity was 20/20 and dilated fundus exam was unremarkable.

Two days after his antibiotic course was completed, the patient presented with redness and right eye pain. Visual acuity was 20/40, intraocular pressure was 20 mmHg, and the patient had 2–3+ cells in the anterior chamber. Concerned for recurrence, an anterior chamber tap was performed, oral trimethoprim-sulfamethoxazole was restarted, and topical gentamicin and prednisolone acetate were increased to every 2 h. Three days later, 4+ anterior chamber cells were noted, and his intraocular pressure was 36 mmHg. He received an intravitreal injection of amikacin. Cultures from the repeat anterior chamber tap showed no growth, and universal bacterial Polymerase Chain Reaction (PCR) was negative.

**Fig. 4:**
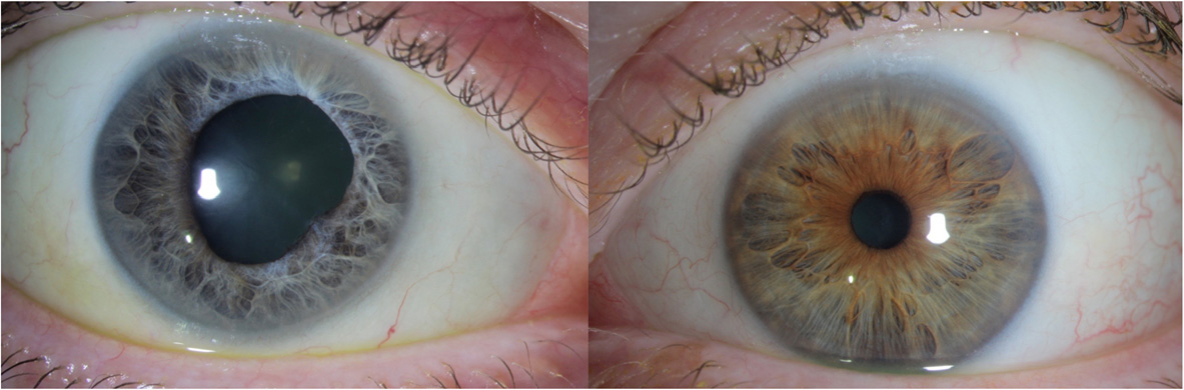
Photos of the right and left eyes show the development of iris heterochromia due to shedding of iris pigment in the right eye, the infected eye

One month later, the patient’s visual acuity had returned to 20/20, intraocular pressure was 14 mmHg, and he was tapered off all medications and drops except trimethoprim-sulfamethoxazole. The patient developed another recurrence of iritis and was restarted on prednisolone acetate 1 %. Given the patient’s episodes of recurrent inflammation, he remains on prophylactic dosing of trimethoprim-sulfamethoxazole and prednisolone acetate 1 % daily. The patient was last seen 11 months after his initial presentation and was doing well with visual acuity 20/20 and on maintenance doses of daily 1 % prednisolone acetate and trimethoprim-sulfamethoxazole three times a week. The patient has developed heterochromia as a result of his *Listeria* endophthalmitis due to shedding of iris pigment in the infected eye (Fig. 4).

### Discussion

Despite adequate antibiotic treatment, numerous case reports show poor visual acuity outcomes following *L. monocytogenes* endophthalmitis. Most patients have final vision worse than 20/100, and one case resulted in phthisis [2, 6, 8, 11–16]. As with all cases of endophthalmitis, prompt intravitreal antibiotics is the key factor for a good visual outcome [9]. At our institution, bacterial endophthalmitis is empirically treated with intravitreal vancomycin and ceftazidime. However, *L. monocytogenes* has variable resistance to both of these agents.

Due to exceedingly rare prevalence of *L. monocytogenes* endophthalmitis, it is not high on most differentials, and diagnosis is made only after culture. This can delay proper antibiotic coverage, thereby allowing a sizeable inflammatory response to mount. Therefore, in the setting of a hypopyon—in particular a dark hypopyon—with high intraocular pressure, *Listeria* should be considered and appropriate antibiotics administered. Given *Listeria* endophthalmitis is most commonly endogenous, systemic antibiotics are an important complement to intravitreal antibiotics.

It is unclear if the patient’s recurrent episodes of inflammation represent repeated infection. Follow-up culture and universal PCR were negative suggesting this may be recurrent inflammation alone. Recurrences of systemic *L. monocytogenes* infections have been documented in adult patients, and recurrence of *L. monocytogenes* infections in the pediatric population has been linked to immune system deficiencies [17–21]. There have not been any previous reports of recurrent *L. monocytogenes* endophthalmitis in the literature.

In this patient, the majority of the inflammatory response was localized to the anterior chamber. After proper antibiotic therapy and oral steroids, the patient had a persistent, large, and dense hypopyon that was not resolving despite clearing of anterior chamber cells. In addition, the hypopyon was causing progressive corneal edema due to the presence of inflammatory mediators in the hypopyon. This led to the decision to pursue anterior chamber washout to prevent further corneal damage.

Ocampo et al. treated recalcitrant anterior segment inflammation in sarcoid uveitis patients with surgical removal of iris granulomas, applying similar logic used to justify vitrectomy in chronic uveitis. By removing immune cells and cytokines, one can halt the cycle of ongoing inflammation and prevent further damage to native structures [22]. Our patient’s vision improved dramatically following anterior chamber washout, and the cornea cleared shortly thereafter. It is our observation from this case and review of the literature that when a robust inflammatory response persists after the initial infectious trigger has been adequately treated, surgical removal of the inflammatory milieu may be indicated.

This case exemplifies the importance of a wide differential diagnosis when approaching endophthalmitis. In endogenous cases, there is a range of potential culprit organisms, as in this case, that may not be responsive to empiric therapy [7]. Patients should be followed closely until a therapeutic response is evident. Continued follow-up even after adequate treatment is equally important in order to promptly treat recurrent inflammation or infection.

## Consent

The patient has given his consent for publication.

## Abbreviation

IOP: intraocular pressurePolymerase Chain Reaction (PCR)
